# Dominance of vaccine serotypes in pediatric invasive pneumococcal infections in Portugal (2012–2015)

**DOI:** 10.1038/s41598-018-36799-x

**Published:** 2019-01-09

**Authors:** Catarina Silva-Costa, Maria J. Brito, Sandra I. Aguiar, Joana P. Lopes, Mário Ramirez, José Melo-Cristino, Portuguese Group for the Study of Streptococcal Infections, Teresa Vaz, Marília Gião, Rui Ferreira, Ana Bruschy Fonseca, Henrique Oliveira, Ana Cristina Silva, Hermínia Costa, Maria Fátima Silva, Maria Amélia Afonso, Margarida Pinto, Odete Chantre, João Marques, Isabel Peres, Isabel Daniel, Ema Canas, Teresa Ferreira, Cristina Marcelo, Lurdes Monteiro, Luís Marques Lito, Filomena Martins, Maria Ana Pessanha, Elsa Gonçalves, Teresa Morais, Teresa Marques, Cristina Toscano, Paulo Lopes, Luísa Felício, Angelina Lameirão, Ana Paula Mota Vieira, Margarida Tomaz, Rosa Bento, Maria Helena Ramos, Ana Paula Castro, Fernando Fonseca, Ana Paula Castro, Graça Ribeiro, Rui Tomé Ribeiro, Celeste Pontes, Luísa Boaventura, Catarina Chaves, Teresa Reis, Nuno Canhoto, Teresa Afonso, Teresa Pina, Helena Peres, Ilse Fontes, Paulo Martinho, Ana Domingos, Gina Marrão, José Grossinho, Manuela Ribeiro, Helena Gonçalves, Alberta Faustino, Adelaide Alves, Maria Cármen Iglesias, Maria Paula Pinheiro, R. Semedo, Adriana Coutinho, Luísa Cabral, Olga Neto, Luísa Sancho, José Diogo, Ana Rodrigues, Isabel Nascimento, Elmano Ramalheira, Fernanda Bessa, Raquel Diaz, Isabel Vale, Ana Carvalho, José Miguel Ribeiro, Maria Antónia Read, Valquíria Alves, Margarida Monteiro, Engrácia Raposo, Maria Lurdes Magalhães, Helena Rochas, Anabela Silva, Margarida Rodrigues, José Mota Freitas, Sandra Vieira, Maria Favila Meneses, José Germano de Sousa, Mariana Bettencourt Viana, Isaura Terra, Vitória Rodrigues, Patrícia Pereira, Jesuína Duarte, Paula Pinto, Ezequiel Moreira, João Ataíde Ferreira, Adília Vicente, Paulo Paixão, Natália Novais, Portuguese Study Group of Invasive Pneumococcal Disease of the Pediatric Infectious Disease Society, Sónia Aires, Cristina Ferreira, Eurico Gaspar, Manuela Ferreira, Fernanda Pereira, Maria José Dinis, Álvaro Sousa, Paulo Teixeira, José Amorim, Cláudia Monteiro, Isabel Carvalho, Sofia Arosa, Margarida Guedes, Laura Marques, Ana Braga, Margarida Tavares, Isabel Cunha, Lurdes Vicente, Maria Manuel Zarcos, Helena Almeida, Silvia Almeida, Fernanda Rodrigues, Cristina Resende, Eulália Afonso, Luísa Mendes, Cristina Faria, Ana Luísa Teixeira, António Mendes, Teresa Tomé, Mónica Rebelo, Filomena Pereira, Gustavo Rodrigues, Alexandra Costa, Ana Teixeira, Sofia Lima, Érica Laima, Maria Ana S. Nunes, Filipa Prata, Pedro Flores, Manuela Brandão, João Calado Nunes, Rosário Massa, Florbela Cunha, Paula Correia, Anabela Brito, João Franco, Cristina Didelet, Estela Veiga, Carla Cruz, Graça Seves, Céu Novais, Maria João Virtuoso, Nancy Guerreiro, Amélia Cavaco, Francisco Gomes, Dora Gomes, Isabel Monteiro

**Affiliations:** 1 Instituto de Microbiologia, Instituto de Medicina Molecular, Faculdade de Medicina, Universidade de Lisboa, Lisboa, Portugal; 2 Centro Hospitalar de Lisboa Central, Lisboa, Portugal; 3 Centro Hospitalar do Barlavento Algarvio, Portimão, Portugal; 4 Hospital de Cascais, Cascais, Portugal; 5 Centro Hospitalar de Coimbra, Coimbra, Portugal; 6 Centro Hospitalar de Entre Douro e Vouga, Santa Maria da Feira, Portugal; 7 Hospital da Figueira da Foz, Figueira da Foz, Portugal; 8 Centro Hospitalar Lisboa Norte, Lisboa, Portugal; 9 Centro Hospitalar Lisboa Ocidental, Lisboa, Portugal; 10 Centro Hospitalar de Vila Nova de Gaia/Espinho, Vila Nova de Gaia e Espinho, Portugal; 11 Centro Hospitalar do Alto Ave, Guimarães, Portugal; 12 Centro Hospitalar do Baixo Alentejo, Beja, Portugal; 13 Centro Hospitalar do Porto, Porto, Portugal; 14 Centro Hospitalar da Póvoa do Varzim/Vila do Conde, Póvoa do Varzim e Vila do Conde, Portugal; 15 Centro Hospitalar de Trás os Montes e Alto Douro, Vila Real e Peso da Régua e Chaves, Chaves, Portugal; 16 Hospitais da Universidade de Coimbra, Coimbra, Portugal; 17 Hospital Central do Funchal, Funchal, Portugal; 18 Hospital Curry Cabral, Lisboa, Portugal; 19 Hospital de Santa Luzia, Elvas, Portugal; 20 Hospital de Santo André, Leiria, Portugal; 21 Hospital de São João, Porto, Portugal; 22 Hospital de Braga, Braga, Portugal; 23 Hospital Dr. José Maria Grande, Portalegre, Portugal; 24 Hospital do Espírito Santo, Évora, Portugal; 25 Hospital dos SAMS, Lisboa, Portugal; 26 Hospital Dr. Fernando da Fonseca, Amadora, Portugal; 27 Hospital Garcia de Orta, Almada, Portugal; 28 Hospital Infante D. Pedro, Aveiro, Portugal; 29 Hospital de São Teotónio, Viseu, Portugal; 30 Hospital Pedro Hispano, Matosinhos, Portugal; 31 Instituto Nacional de Saúde Ricardo Jorge, Porto, Portugal; 32 Hospital Reynaldo dos Santos, Vila Franca de Xira, Portugal; 33 Centro Hospitalar do Alto Minho, Ponte de Lima e Viana do Castelo, Portugal; 34 Hospital CUF Descobertas, Lisboa, Portugal; 35 Centro Hospitalar do Tâmega e Sousa, Amarante e Guilhufe, Portugal; 36 Hospital Beatriz Ângelo, Loures, Portugal; 37 Centro Hospitalar de Setúbal, Setúbal, Portugal; 38 Hospital Distrital de Santarém, Santarém, Portugal; 39 Centro Hospitalar do Médio Ave, Santo Tirso e Vila Nova de Famalicão, Famalicão, Portugal; 40 Hospital de Faro, Faro, Portugal; 41 Centro Hospitalar do Oeste Norte, Caldas da Rainha, Portugal; 42 Hospital da Luz, Lisboa, Portugal; 43 Hospital da Figueira da Foz, Figueira da Foz, Portugal; 44 Centro Hospitalar do Nordeste, Bragança, Macedo de Cavaleiros e Mirandela, Portugal; 45 Hospital Amato Lusitano, Castelo Branco, Portugal; 46 Centro Hospitalar da Cova da Beira, Covilhã, Portugal; 47 Hospital Sousa Martins, Guarda, Portugal; 48 IPO, Lisboa, Portugal; 49 Hospital Lusíadas, Lisboa, Portugal; 50 Hospital Cruz Vermelha, Lisboa, Portugal; 51 Centro Hospitalar do Médio Tejo, Abrantes, Portugal; 52 Centro Hospitalar do Barreiro Montijo, Barreiro Montijo, Portugal; 53 Hospital de Santo Espírito, Angra do Heroísmo, Portugal; 54 Hospital da Horta, Horta, Portugal; 55 Hospital do Divino Espírito Santo, Ponta Delgada, Portugal

## Abstract

We evaluated the impact of continued 13-valent pneumococcal conjugate vaccine (PCV13) use in the private market (uptake of 61%) in pediatric invasive pneumococcal disease (pIPD) in Portugal (2012–2015). The most frequently detected serotypes were: 3 (n = 32, 13.8%), 14 (n = 23, 9.9%), 1 (n = 23, 9.9%), 7F (n = 15, 6.4%), 19A (n = 13, 5.6%), 6B and 15B/C (both n = 12, 5.2%), and 24F, 10A and 12B (all with n = 10, 4.3%). Taken together, non-PCV13 serotypes were responsible for 42.2% of pIPD with a known serotype. The use of PCR to detect and serotype pneumococci in both pleural and cerebrospinal fluid samples contributed to 18.1% (n = 47) of all pIPD. Serotype 3 was mostly detected by PCR (n = 21/32, 65.6%) and resulted from a relevant number of vaccine failures. The incidence of pIPD varied in the different age groups but without a clear trend. There were no obvious declines of the incidence of pIPD due to serotypes included in any of the PCVs, and PCV13 serotypes still accounted for the majority of pIPD (57.8%). Our study indicates that a higher vaccination uptake may be necessary to realize the full benefits of PCVs, even after 15 years of moderate use, and highlights the importance of using molecular methods in pIPD surveillance, since these can lead to substantially increased case ascertainment and identification of particular serotypes as causes of pIPD.

## Introduction

The introduction of pneumococcal conjugate vaccines (PCVs) into routine infant immunization programs worldwide has led to significant decreases in overall incidence of invasive pneumococcal disease (IPD), both in children and in adults^1–5^. Moreover, massive changes in the distribution of *Streptococcus pneumoniae* serotypes were also noted, with major decreases in the incidence of IPD caused by vaccine serotypes (VTs), i.e. those included in PCV formulations^1,2,6,7^. However, this was accompanied in some cases by an increase in the incidence of IPD due to non-vaccine serotypes (NVTs)^5,8,9^. Additionally, antimicrobial susceptibility may also be affected by the use of PCVs since these target serotypes frequently associated with antimicrobial resistance^10,11^.

In Portugal, although available only in the private market, the introduction of the 7-valent pneumococcal conjugate vaccine (PCV7) in 2001 led to significant changes in the serotypes responsible for pediatric IPD (pIPD)^2^. Among the most relevant changes was the rise of non-PCV7 serotypes 1, 7F and 19A as major causes of pIPD^12^. During this period uptake peaked at 75% around 2008^1^. In mid-2009, PCV10 became available, including PCV7 serotypes and additionally serotypes 1, 5 and 7F. Soon after this, in early 2010, PCV13 was introduced in Portugal, further including serotypes 3, 6A and 19A, and became the leading vaccine used in Portugal.

Despite a decline in PCV uptake to 62% in 2012, which remained stable until 2014 at 61%^13^, the introduction of higher valency vaccines was accompanied by declines of pIPD incidence, with a decrease from 8.19 cases per 100,000 inhabitants in 2008–2009 to 4.52 per 100,000 inhabitants in 2011–2012^1^. Despite PCV7 use since 2001, this decrease was mainly due to decreases in the number of cases due to serotypes included in the newest vaccines, particularly serotype 1 and 19A in older and younger children, respectively, and not to further reductions in the number of cases due to PCV7 serotypes, including in children under 2 years of age. That study also suggested that some of the serotype changes may not have been triggered by vaccination^1^, emphasizing the importance of the natural fluctuations of serotypes and the need to perform continuous epidemiological surveillance.

In July 2015, PCV13 was introduced in the National Immunization Plan (NIP), for children born after January 2015, with doses given at 2, 4 and 12 months of age. The aims of this work were to determine the incidence of pIPD in Portugal, as well as serotype distribution and susceptibility patterns among the pneumococcal population between July 2012 and June 2015, just prior to the introduction of PCV13 in the NIP.

## Results

### Isolate collection

**Figure 1:**
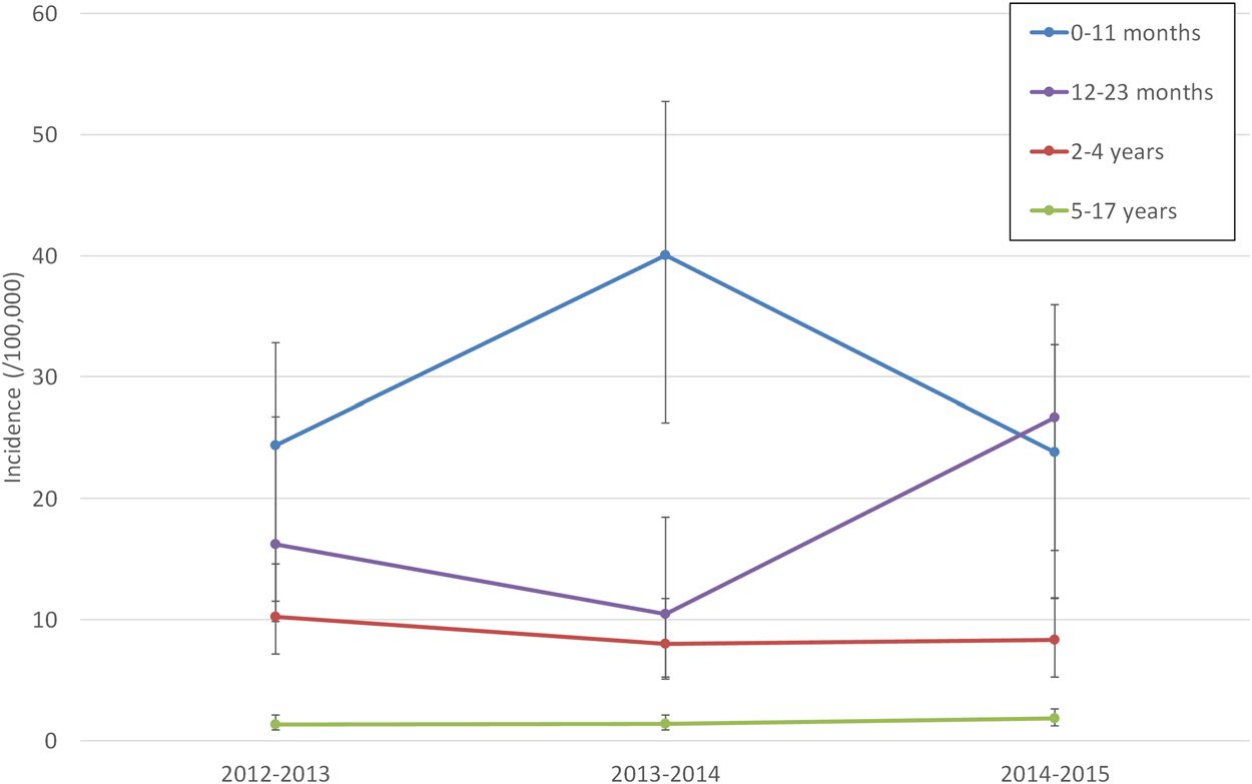
Incidence of invasive pneumococcal disease in children and adolescents in Portugal (2012–2013 to 2014–2015). The 95% confidence intervals for the incidence estimates are indicated.

Between July 2012 and June 2015, a total of 259 cases of pIPD were reported. Table 1 summarizes their distribution by age and epidemiological year while Fig. 1 presents the annual incidence of pIPD by age group. We found no temporal and geographic clustering of cases which could suggest the existence of outbreaks. Although there were variations in the incidence of pIPD in all age groups these did not show clear trends (Table S1). For 27 cases (10.4%) neither the isolate nor a patient sample was sent to the central laboratory for characterization. A total of 185 isolates (79.7%) and 47 samples (20.2%) of LCR (n = 6) or pleural fluid (n = 41) positive for *S. pneumoniae* were available for further characterization. Available isolates were recovered from blood (n = 149, 80.6%), CSF (n = 25, 13.4%), pleural fluid (n = 7, 3.8%), and synovial fluid (n = 4, 2.2%).

**Table 1:** Cases and missing samples from invasive pneumococcal disease from patients <18 yrs, Portugal, July 2012–June 2015.

Epidemiological years^a^	Cases/number of missing samples, by age group				
	0–11 months	12–23 months	2–4 years	5–17 years	Total
2012–2013	21/2	15/2	30/2	19/3	**85/9**
2013–2014	33/5	9/1	23/0	19/1	**84/7**
2014–2015	20/3	22/2	23/2	25/4	**90/11**
**Total**	**74/10**	**46/5**	**76/4**	**63/8**	**259/27**

^a^From week 26 of one year to week 25 of the following year.

### Serotype distribution

**Figure 2:**
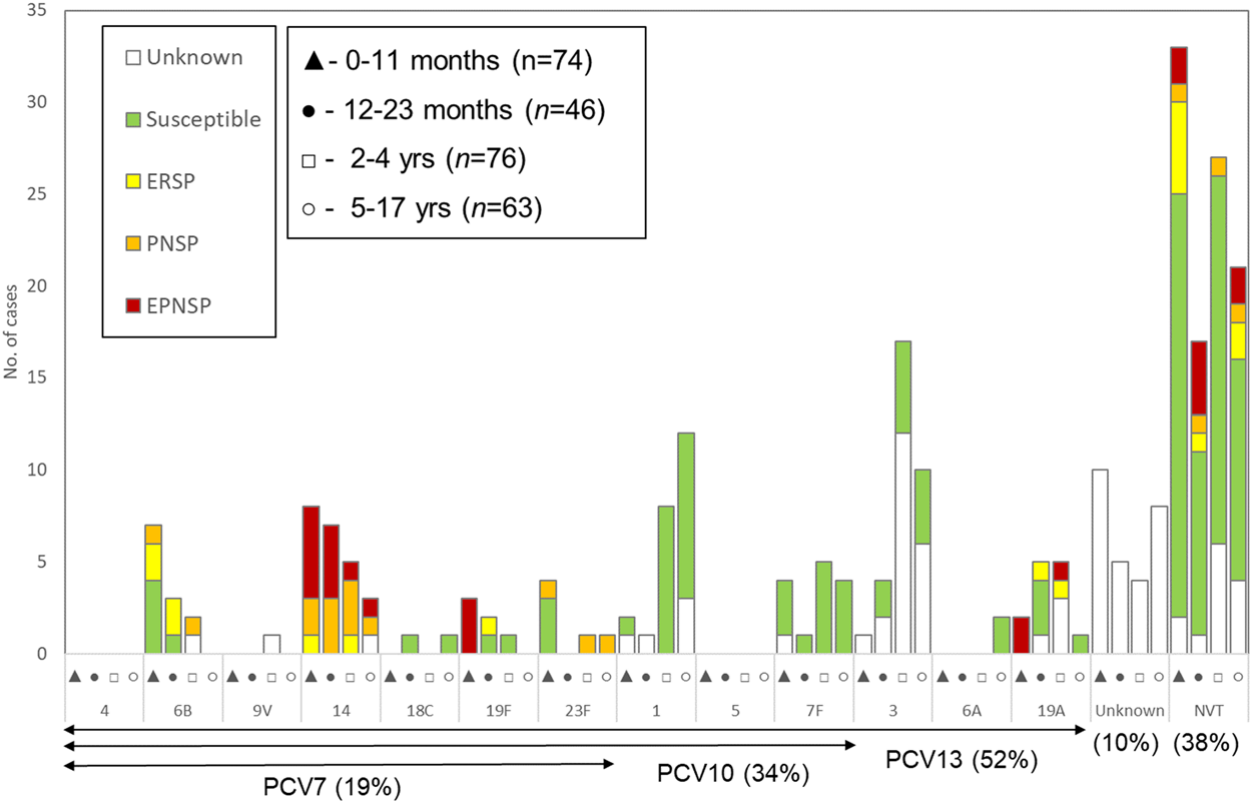
Number of samples representing serotypes present in conjugate vaccines causing invasive infections in Portugal (2012–2013 to 2014–2015). The number of samples representing each serotype in each of the age groups considered is indicated. Isolates presenting both erythromycin resistance and penicillin non-susceptibility (EPNSP) are represented by red bars. Penicillin non-susceptible isolates (PNSP) are indicated by orange bars. Erythromycin resistant isolates (ERSP) are indicated by yellow bars. Isolates susceptible to both penicillin and erythromycin are represented by green bars. Cases where no sample was available or where pneumococci were detected exclusively by PCR and for which susceptibility is unknown, are indicated by white bars. The serotypes included in each of the conjugate vaccines are indicated by the arrows. NVT – non-vaccine serotypes, i.e., serotypes not included in any of the currently available conjugate vaccines (PCV7, PCV10 and PCV13). The values indicated below the arrows are the proportion of each group in the overall cases (n = 259).

**Figure 3:**
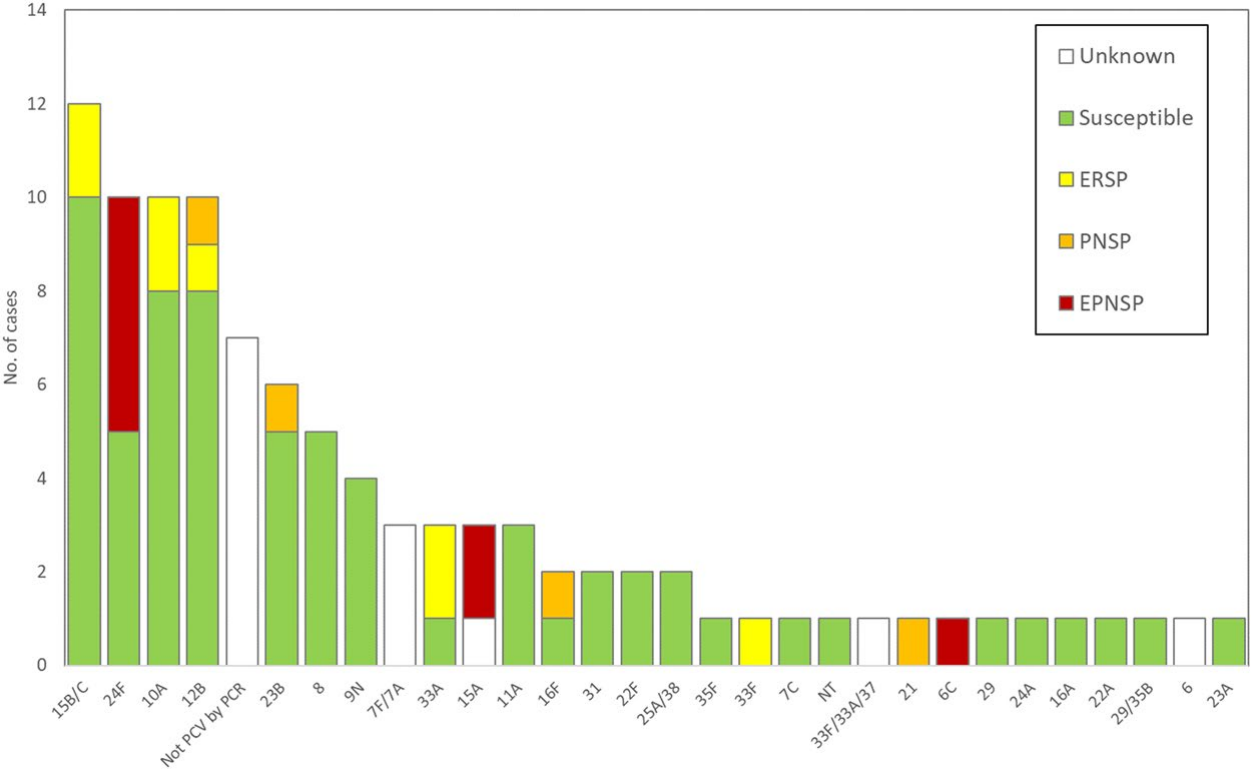
Number of samples representing serotypes not present in conjugate vaccines causing invasive infections in Portugal (2012–2013 to 2014–2015). See the legend of Fig. 2. NT – Non-typeable. Some serotypes could not be unambiguously determined and are indicates as such (see text). Among the 12 isolates identified as 15B/C, 8 were originally typed as 15B and 4 isolates were typed as 15C.

Among the 232 isolates and patient samples 39 different capsular types were detected, including samples for which the serotype could not be unambiguously determined– 7F/7A (n = 3), 25A/38 (n = 2), serogroup 6 (n = 1), 33F/33A/37 (n = 1), 29/35B (n = 1), as well as non-typable isolates (Figs 2 and 3). This translated into a high serotype diversity (SID = 0.943; CI95% 0.933 to 0.954). The most frequent serotypes, which accounted for 69% of all pIPD in the study period, were serotypes 3 (n = 32, 13.8%), 14 (n = 23, 9.9%), 1 (n = 23, 9.9%), 7F (n = 15, 6.4%), 19A (n = 13, 5.6%), 6B and 15B/C (both n = 12, 5.2%), and 24F, 10A and 12B (all with n = 10, 4.3%). Overall pIPD due to the serotypes included in PCV13 remained significant, accounting for 57.8% (21.6% PCV7) of the isolates and patient samples. When considering individual serotypes (n > 5) in all age groups, we noted significant decreases in the number of cases from 2012–2013 to 2014–2015 of serotypes 1 and 15B/C, and an increase of serotype 14, but none of these changes was statistically supported (Table 2).

**Table 2:** Serotypes responsible for ≥10 IPD cases with a known serotype.

Serotype	Number of isolates^a^		
	2012–2013	2013–2014	2014–2015
1	12	5	6
3	12	10	10
6B	5	4	3
7F^b^	5	3	6
10 A	3	5	2
12B	0	8	2
14	5	6	12
15B/C	7	3	2
19 A	5	1	7
24 F	5	2	3

^a^Epidemiological years: from week 26 of one year to week 25 of the following year.

^b^The three cases in which the determined serotype was 7 F/7A are not included.

**Figure 4:**
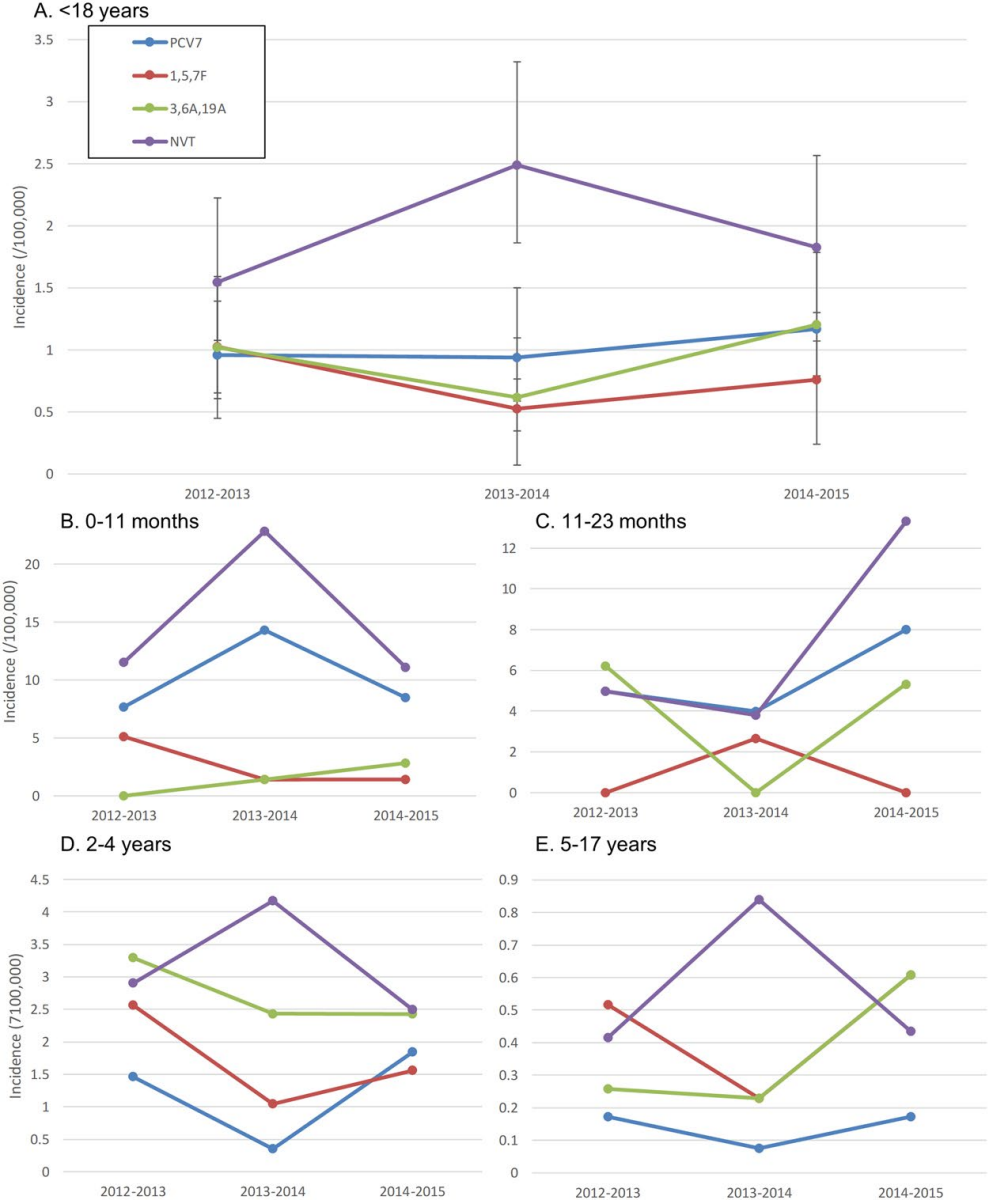
Incidence of invasive pneumococcal disease in children and adolescents in Portugal (2011–2012 to 2014–2015). NVT – non-vaccine serotypes.

To estimate the incidence of pIPD due to individual serotypes, the cases for which no isolate was available were assumed to have the same serotype distribution as that found among isolates from the same epidemiological year and age group. Since the molecular serotyping technique used on culture negative samples comprised all the serotypes included in PCV13, when a serotype could not be determined by this method, the case was considered caused by an NVT serotype. Cases whose serotype remained undetermined (7F/7A, 25A/38, serogroup 6, 33F/33A/37, 29/35B) and non-typable isolates were considered NVTs. As with overall pIPD incidence, in some age groups there were variations in the incidence of pIPD due to the serotypes included in each of the conjugate vaccine formulations, but these showed no clear trend (Fig. 4).

### Antimicrobial susceptibility

Susceptibility to antimicrobials was tested among the 185 available isolates and is summarized in Figs 2 and 3 and Table 3.

**Table 3:** Antimicrobial resistance of *Streptococcus pneumoniae* isolates responsible for invasive disease in patients <18 yrs, Portugal, July 2012–June 2015 (n = 185).

Antibiotic^a^	Number of resistant isolates (%)^b^			
	0–11 months (n = 59)	12–23 months (n = 36)	2–4 years (n = 49)	5–17 years (n = 41)
PEN^c^	17 (28.8)	12 (33.3)	8 (16.3)	6 (14.6)
MIC_90_	1.5	1.5	1	0.094
MIC_50_	0.016	0.016	0.016	0.016
CRO^c^	4 (6.8)	1 (2.8)	0 (0)	0 (0)
MIC_90_	1	1	0.75	0.064
MIC_50_	0.02	0.023	0.016	0.016
CTX^c^	2 (3.4)	2 (5.6)	0 (0)	0 (0)
MIC_90_	0.75	0.75	0.5	0.094
MIC_50_	0.02	0.02	0.02	0.02
ERY	20 (33.9)	13 (36.1)	4 (8.2)	5 (12.2)
CLI	16 (27.1)	11 (30.6)	3 (6.1)	5 (12.2)
CHL	2 (3.4)	1 (2.8)	2 (4.1)	0 (0)
SXT	16 (27.1)	5 (13.9)	8 (16.3)	7 (17.1)
TET	12 (20.3)	5 (13.9)	4 (8.2)	3 (7.3)
VAN	0 (0)	0 (0)	0 (0)	0 (0)
LVX	0 (0)	0 (0)	0 (0)	0 (0)
LZD	0 (0)	0 (0)	0 (0)	0 (0)

^a^CHL: chloramphenicol; CLI: clindamycin; CRO: ceftriaxone; CTX: cefotaxime; ERY: erythromycin; LVX: levofloxacin; LZD: linezolid; PEN: penicillin; SXT: trimethoprim-sulfamethoxazole; TET: tetracycline; VAN: vancomycin.

^b^Unless otherwise specified.

^c^Number and percentage of non-susceptible isolates is indicated.

Overall 43 isolates (23.2%) were nonsusceptible to penicillin (PNSP) – 34 (18.4%) expressed low-level resistance and 9 (4.8%) high-level resistance. Considering the current CLSI breakpoints for parenteral penicillin^14^, 6 isolates (24%) from the CSF would have been considered resistant and 6 isolates (3.8%) from non-meningitis cases would have been considered nonsusceptible, all intermediately resistant. Resistance to erythromycin (ERP) was found in 42 isolates (22.7%), of which 35 isolates (83.3%) expressed the constitutive MLS_B_ phenotype and 7 (16.7%) the M phenotype. All isolates were susceptible to levofloxacin, vancomycin and linezolid. The simultaneous expression of erythromycin resistance and penicillin non-susceptibility (EPNSP) was found in 13.5% of the isolates. There were no significant trends in resistance within the study period.

Serotype 14 contributed greatly to erythromycin resistance (31.0%) and to penicillin nonsusceptibility (46.5%) (Fig. 2). Serotypes included in PCV7 represented 77.8%, 41.1% and 56.0% of PNSP, ERSP and EPNSP, respectively, while serotypes included in PCV13 constituted 77.8%, 52.9% and 68.0%, respectively.

## Discussion

pIPD incidence varied only modestly and without a clear trend in 2012–2015, in contrast to the significant declines in pIPD incidence seen in 2008–2012, particularly among the younger groups^1^. As was seen previously, PCV7 serotypes remain important causes of pIPD (with incidences varying between 0.94–1.12 cases/100,000 in 2012–2015), with the slower uptake and lower vaccination coverage reached in Portugal, when compared to countries where PCV7 was introduced in the NIP, and the high resistance of isolates expressing PCV7 serotypes, particularly to penicillin and the macrolides (77% of all PNSP and 41% of all ERP), possibly being important factors for their persistence^1^.

The serotype dynamics were different in the various age groups, but the dominant feature of the 2012–2015 period was serotype instability, with the incidence of the serotypes included in the different PCVs and NVTs fluctuating without a definite trend in the various age groups. This may be due to the use of PCVs outside of the NIP and the existence of a considerable number of unvaccinated children that remain susceptible to VT disease. The variable yearly dissemination of PCV serotypes in the latter group could account for some of the fluctuations seen. In neighboring Spain, the switch in Madrid from administering PCV13 within the NIP to the private sector, with a concomitant decline in uptake from 95% to 67–73% (similar to the one in Portugal), led to a stagnation in the decline of PCV13 pIPD^15^, suggesting that a high vaccination uptake is needed to reap the full benefits of PCV use seen elsewhere^16,17^.

Although the number of pIPD cases identified solely by molecular methods was not very different from that in 2008–2012^1^, the proportion was higher in 2012–2015 (n = 41/471 = 8.6% in 2008–2012 versus n = 47/259 = 18.1% in 2012–2015). In contrast to our last study, in this period we serotyped by molecular methods samples from which no isolate was recovered. This resulted in a significant improvement in the identification of the serotypes causing complicated pneumonias, in which pneumococci were frequently identified exclusively by molecular methods in pleural fluid, and which constituted 20.2% of all samples with serotype information. Serotype 3, the most frequently detected serotype in 2012–2015 (Fig. 2 and Table 2) was predominantly detected in pleural fluid samples by molecular methods (n = 21/32, 65.6%) and, as reported and discussed elsewhere^13^, was frequently associated with vaccine failures. The persistence of serotype 3 despite vaccination and its association with complicated pneumonia, together with the decline of other vaccine serotypes associated with other disease presentations and positive cultures, is potentially contributing to the increase in the relative importance of molecular methods in identifying pIPD cases. Serotype 3 was not among the most prevalent serotypes in previous years^1,2,12^, but this would have also been the case in this study if molecular methods had not been used for serotyping directly from culture negative patient samples. A recent study from England and Wales looking at IPD in all age groups, reported that PCR-confirmed cases were a minority of all IPD (<4%) and were therefore excluded from the analysis performed by the authors^17^. However, in our context molecular methods did contribute for the identification of a significant fraction of all pIPD cases in children and the dominance of particular serotypes in complicated pneumonia cases can have important consequences for the overall serotype distribution of IPD in children, as seen here.

The most important NVTs were 15B/C, 10A, 12B and 24F. Most of these serotypes (15B/C, 10A and 24F) were also among the most prevalent causes of post-PCV13 IPD in several other countries^16^ and were associated with cases of pIPD in Portugal in 2008–2012^1^. In contrast, serotype 12B was not found among the prevalent serotypes elsewhere and was detected only once in pIPD in Portugal in 2008–2012^1^, suggesting a possible recent emergence of a particularly virulent lineage.

The PCV7 serotypes, particularly serotype 14, remained the most important serotypes responsible for antimicrobial resistance, suggesting that this may be a factor driving their persistence. Relative to the previous period^1^ resistance decreased only modestly, despite the sharp decrease of serotype 19A, a PCV13 serotype which was associated with resistance. Asymptomatic carriage with PCV13 serotypes was common in Portugal before the use of PCV13, although it was subject to temporal fluctuations^18^. However, the efficacy of vaccines in eradicating colonization, and therefore in reducing exposure, is known to vary between vaccine serotypes. For instance, despite being targeted by all PCVs available to date, serotype 19F remained common in nasopharyngeal carriage in children in Portugal in the late post-PCV7 period^19^ and in the post-PCV13 period^20^, including among vaccinated children. Such differences may mean that the overall exposure of children to certain serotypes is not significantly changed, potentially playing an important role in their persistence as causes of pIPD among both vaccinated and unvaccinated children. Besides their continued circulation in carriage, another important factor to consider is the different invasive disease potential of distinct serotypes, measuring their propensity to cause pIPD. In the case of serotypes 19A and 14, only the latter was shown to have an enhanced invasive disease potential when taken as a whole, although lineages with variable invasive disease potential were found within both serotypes^21^.

Our study has several limitations. The study was not designed to collect information important to assess the severity of the infections caused by the different serotypes (e.g. hospitalization, ICU admission, 30-day mortality) nor the vaccination status of the cases, which would be important to identify vaccine failures. We believe that few cases of confirmed pIPD were diagnosed outside of our network since the criteria for the identification of a pIPD case are the isolation of pneumococci or the identification of pneumococcal DNA in normally sterile fluids, tests which are almost exclusively performed in hospital laboratories. The stability of our surveillance network, the active nature of the surveillance and the involvement of both pediatric and microbiology departments of many hospitals covering the entire country, further substantiates the identification of most cases by our surveillance. We cannot guarantee that the serotype distribution of the cases where isolates were unavailable followed the serotype distribution of available isolates. However, the small proportion of cases with unknown serotype information ensures that our extrapolation will not greatly affect the results.

Following the substantial declines in PCV13 pIPD seen previously^1^, these serotypes have not declined further and remain the most important causes of pIPD (Fig. 2). The persistence of these serotypes, including the PCV7 serotypes that have been subject to vaccine pressure for more than a decade, suggests that their persistence could be due in part to the relatively moderate vaccination uptake in Portugal. Uptake reached 75% around 2008^1^ but declined to 61% in recent years^13^, a significantly lower uptake than in countries where PCVs are in the NIP and highlighting the potential benefits of increasing vaccination uptake. Continued surveillance will monitor the extent of VT resilience and will clarify if the vaccine failures seen with serotype 3 and its rise to most important pIPD serotype will be maintained following the introduction of PCV13 in the NIP, when a high vaccine uptake (≥95%) is expected.

## Materials and Methods

### Bacterial isolates

Since 2007, the Portuguese Group for the Study of Streptococcal Infections and the Portuguese Study Group of Invasive Pneumococcal Disease of the Pediatric Infectious Disease Society have monitored pneumococcal invasive infections in Portugal. The study was approved by the Institutional Review Board of the Centro Académico de Medicina de Lisboa. These were considered surveillance activities and were exempt from informed consent. All methods were performed in accordance with the relevant guidelines and regulations. The data and isolates were de-identified so that these were irretrievably unlinked to an identifiable person. During the surveillance period this involved the microbiology laboratories and pediatric departments of 55 hospitals throughout Portugal. All centers reported during the entire period. A case of IPD was defined as a person from whom an isolate of *S. pneumoniae* was recovered from a normally sterile body site (not including middle ear fluid) or from whom pneumococcal DNA was detected in cerebrospinal fluid (CSF) or pleural fluid. Isolates recovered up to June 2012 were previously characterized^1,2,12,22^. Only isolates recovered from pediatric patients (<18 years) and recovered between July 2012 (week 26) and June 2015 (week 25) were included in the present study. Data on pleural fluid samples was reported previously^13^. Epidemiological years were defined as spanning from week 26 to week 25 of the following year. Only one isolate from each patient in a 90-day interval was included. All strains were identified as *S. pneumoniae* by colony morphology and hemolysis on blood agar plates, optochin susceptibility and bile solubility. In the case of body fluids where the detection of pneumococci was performed by molecular methods, two *S. pneumoniae* genes (*lytA* and *wzg*) were used for bacterial identification^13^. Incidences were calculated based on the entire Portuguese population of the relevant age groups using data available from the first calendar year of each epidemiological year from “Instituto Nacional de Estatística” (www.ine.pt). This calculation assumes that all pIPD cases are treated within the 55 hospitals in our network.

### Serotyping and antimicrobial susceptibility testing

Serotyping was performed by the standard capsular reaction test using the chessboard system and specific sera (Statens Serum Institut, Copenhagen, Denmark). In cases where the diagnosis was done by molecular methods, the serotypes were also determined by PCR with a reaction targeting 21 serotypes^13^. Serotypes were classified into vaccine serotypes (VT), i.e., those included in PCV7 (serotypes 4, 6B, 9V, 14, 18C, 19F, 23F), the additional three found in PCV10 relative to PCV7 (addPCV10: 1, 5, 7F), the additional three found in PCV13 relative to PCV10 (addPCV13: 3, 6A, 19A), and non-vaccine serotypes (NVT). Since the PCR reaction includes all PCV13 serotypes, when a serotype could not be identified this was grouped into the NVT group. Etest strips (AB Biodisk, Solna, Sweden) were used to determine the minimal inhibitory concentrations (MICs) for penicillin, cefotaxime, ceftriaxone and levofloxacin. In 2008, the Clinical and Laboratory Standards Institute (CLSI) changed the recommended breakpoints to those currently used to interpret MIC values^14^. Unless otherwise stated, we used the CLSI-recommended breakpoints prior to 2008^23^ as epidemiological breakpoints, allowing the comparison with previous studies.

Isolates were further characterized by determining their susceptibility to erythromycin, clindamycin, vancomycin, linezolid, tetracycline, trimethroprim-sulfamethoxazole and chloramphenicol by the Kirby-Bauer disk diffusion technique, according to the CLSI recommendations and interpretative criteria^14^. Macrolide resistance phenotypes were identified using a double disc test with erythromycin and clindamycin. Simultaneous resistance to erythromycin and clindamycin defines the MLS_B_ phenotype (resistance to macrolides, lincosamides and streptogramin B) while non-susceptibility only to erythromycin indicates the M phenotype.

### Statistical analysis

Simpson’s index of diversity (SID) and respective 95% confidence intervals (CI95%) was used to measure serotype diversity^24^. The Cochran-Armitage test was used for trends with the false discovery rate (FDR) correction for multiple testing^25^. A p < 0.05 was considered significant for all tests.

## Electronic supplementary material

Table S1. Incidence of invasive pneumococcal infections caused by Streptococcus pneumoniae serotypes included in the conjugate vaccine formulations by age group, Portugal, July 2012-June 2015

## Data Availability

The datasets generated during the current study are available from the corresponding author on reasonable request.
