# Supplementary material for: Dominance of vaccine serotypes in pediatric invasive pneumococcal infections in Portugal (2012–2015)
Source: Sci Rep. 2019 Jan 9;9:6. doi: 10.1038/s41598-018-36799-x (PMC6327022; doi:10.1038/s41598-018-36799-x)
Supplement: Supplementary file 1 — Table S1. Incidence of invasive pneumococcal infections caused by Streptococcus pneumoniae serotypes included in the conjugate vaccine formulations by age group, Portugal, July 2012-June 2015 [file 41598_2018_36799_MOESM1_ESM.pdf]

Dominance of vaccine serotypes in pediatric invasive pneumococcal infections in Portugal (2012-2015)

Catarina Silva-Costa[1]; Maria J. Brito[2]; Sandra I. Aguiar[1]; Joana P. Lopes, Mário Ramirez*; José Melo-Cristino[1], on behalf of the Portuguese Group for the Study of Streptococcal Infections and the Portuguese Study Group of Invasive Pneumococcal Disease of the Pediatric Infectious Disease Society.

[1]Instituto de Microbiologia, Instituto de Medicina Molecular, Faculdade de Medicina, Universidade de Lisboa, Lisboa, Portugal; [2]Centro Hospitalar de Lisboa Central, Lisboa, Portugal.

Table S1. Incidence of invasive pneumococcal infections caused by *Streptococcus pneumoniae* serotypes included in the conjugate vaccine formulations by age group, Portugal, July 2012-June 2015.

Age group	Serotype group[a]	Incidence (95% confidence intervals)[b]		
		2012-2013	2012-20132	2014-2015
[0,17]	PCV7	0.96 (0.61 - 1.52)	0.94 (0.59 - 1.5)	1.17 (0.77 - 1.78)
	PCV10	1.99 (1.44 - 2.74)	1.46 (1 - 2.13)	1.93 (1.39 - 2.69)
	PCV13	3.01 (2.32 - 3.9)	2.08 (1.52 - 2.85)	3.13 (2.42 - 4.06)
	NVT	1.55 (1.08 - 2.22)	2.49 (1.86 - 3.32)	1.83 (1.3 - 2.57)
	All serotypes	4.55 (3.68 - 5.63)	4.57 (3.64 - 5.57)	4.96 (3.92 - 5.93)
[5,17]	PCV7	0.17 (0.05 - 0.57)	0.08 (0.01 - 0.42)	0.17 (0.05 - 0.58)
	PCV10	0.69 (0.37 - 1.28)	0.31 (0.12 - 0.77)	0.78 (0.43 - 1.41)
	PCV13	0.95 (0.56 - 1.61)	0.53 (0.26 - 1.08)	1.39 (0.89 - 2.17)
	NVT	0.42 (0.19 - 0.92)	0.84 (0.48 - 1.48)	0.43 (0.2 - 0.95)
	All serotypes	1.36 (0.87 - 2.13)	1.37 (0.87 - 2.13)	1.82 (1.21 - 2.65)
[2,4]	PCV7	1.46 (0.59 - 3.65)	0.35 (0.06 - 1.97)	1.84 (0.79 - 4.28)
	PCV10	4.03 (2.29 - 7.07)	1.39 (0.54 - 3.57)	3.4 (1.81 - 6.37)
	PCV13	7.32 (4.81 - 11.14)	3.82 (2.13 - 6.84)	5.82 (3.59 - 9.44)
	NVT	2.9 (1.5 - 5.61)	4.17 (2.38 - 7.28)	2.49 (1.2 - 5.18)
	All serotypes	10.22 (7.16 - 14.59)	7.99 (5.22 - 11.76)	8.31 (5.22 - 11.76)
[1,2]	PCV7	4.98 (2.06 - 12.05)	3.98 (1.44 - 10.97)	7.99 (3.79 - 16.85)
	PCV10	4.98 (2.06 - 12.05)	6.63 (2.98 - 14.74)	7.99 (3.79 - 16.85)
	PCV13	11.21 (6.16 - 20.41)	6.63 (2.98 - 14.74)	13.32 (7.44 - 23.86)
	NVT	4.98 (2.06 - 12.05)	3.81 (1.36 - 10.73)	13.32 (7.44 - 23.86)
	All serotypes	16.19 (9.81 - 26.71)	10.44 (5.11 - 18.46)	26.65 (15.68 - 35.95)
[0,1]	PCV7	7.7 (3.66 - 16.2)	14.29 (8.14 - 25.1)	8.49 (4.14 - 17.42)
	PCV10	12.83 (7.18 - 22.95)	15.72 (9.18 - 26.92)	9.91 (5.08 - 19.31)
	PCV13	12.83 (7.18 - 22.95)	17.15 (10.24 - 28.71)	12.74 (7.06 - 23)
	NVT	11.55 (6.26 - 21.29)	22.87 (14.62 - 35.77)	11.1 (5.9 - 20.88)
	All serotypes	24.38 (15.95 - 37.27)	40.02 (27.28 - 53.8)	23.84 (15.03 - 35.87)

[a] PCV – pneumococcal conjugate vaccine, NVT – non-vaccine serotype

[b] Number of cases per 100,000 in specified age group. Epidemiological years: from week 26 of one year to week 25 of the following year.
